# Prediction of Peptide Reactivity with Human IVIg through a Knowledge-Based Approach

**DOI:** 10.1371/journal.pone.0023616

**Published:** 2011-08-24

**Authors:** Nicola Barbarini, Alessandra Tiengo, Riccardo Bellazzi

**Affiliations:** Laboratory of Biomedical Informatics “Mario Stefanelli”, Department of Computer Engineering and Systems Science, University of Pavia, Pavia, Italy; Center for Genomic Regulation, Spain

## Abstract

The prediction of antibody-protein (antigen) interactions is very difficult due to the huge variability that characterizes the structure of the antibodies. The region of the antigen bound to the antibodies is called epitope. Experimental data indicate that many antibodies react with a panel of distinct epitopes (positive reaction). The Challenge 1 of DREAM5 aims at understanding whether there exists rules for predicting the reactivity of a peptide/epitope, i.e., its capability to bind to human antibodies. DREAM 5 provided a training set of peptides with experimentally identified high and low reactivities to human antibodies. On the basis of this training set, the participants to the challenge were asked to develop a predictive model of reactivity. A test set was then provided to evaluate the performance of the model implemented so far.

We developed a logistic regression model to predict the peptide reactivity, by facing the challenge as a machine learning problem. The initial features have been generated on the basis of the available knowledge and the information reported in the dataset. Our predictive model had the second best performance of the challenge. We also developed a method, based on a clustering approach, able to “in-silico” generate a list of positive and negative new peptide sequences, as requested by the DREAM5 “bonus round” additional challenge.

The paper describes the developed model and its results in terms of reactivity prediction, and highlights some open issues concerning the propensity of a peptide to react with human antibodies.

## Introduction

Given their key role in the immune response, antibody-protein interactions play a major role in a variety of clinical domains (infectious diseases, autoimmune diseases, oncology, vaccination and therapeutic interventions). For this reason, the prediction of antibody-protein interactions can be of critical importance [1]–[2]. The antibodies have a wide range of heterogeneous structures generated by genomic recombination: the number of human antibodies is estimated to be around 10^10^ and 10^12^ [3]. The antibodies interact with proteins (called antigens) through their binding sites (called paratopes).

The region of the antigen bound with the paratope is called epitope. Two types of epitopes are typically distinguished in protein-antibody interaction studies: conformational and linear epitopes. A linear/sequential epitope is recognized by its linear sequence of amino acids (primary structure). In contrast, most antibodies recognize conformational epitopes with a specific three-dimensional structure.

All potential linear epitopes of a protein are short peptides that can be synthesized and arrayed on solid supports, e.g. glass slides [4]. By incubating these peptide arrays with antibody mixtures, such as human serum or plasma, it is possible to determine specific interactions between antibodies and peptides.

The binding site of a linear epitope has a typical length ranging between 8 and 10 amino acids. An antibody binds to its epitope/peptide independently of the physical position of the binding site within the peptide. Every amino acid has a different impact on the epitope reactivity; this is not only due to its physicochemical properties but also to its interaction with the neighboring residues within the whole peptide sequence.

It has been often assumed that a specific antibody selectively binds to a specific sequence. However, experimental data indicate that many antibodies bind to a panel of related (or even distinct) peptides with different affinities. The open question is whether there exist rules that enable the prediction of common peptide/epitope sequences, which can be recognized by human antibodies.

In order to address this problem, the DREAM (Dialogue for Reverse Engineering Assessments and Methods) Consortium issued the Epitope-Antibody Recognition (EAR) Specificity Prediction Challenge (Challenge 1). In the experimental work leading to this challenge, 75534 peptides were incubated with commercially available intravenous immunoglobulin (IVIg) fractions. IVIg is a mixture of naturally occurring human antibodies isolated from up to 100000 healthy individuals. From this dataset, high-confidence negative and positive pools of peptides were determined. Training and test datasets were assembled from these peptide pools. The epitope-antibody recognition challenge consists of determining whether each peptide in the test set belongs to the positive or negative set starting from the data of the training set.

A so-called “bonus round” was proposed beside this main challenge. It consists of generating “in-silico” a list of positive and negative new peptide sequences, which should significantly differ from the ones contained in the training set. The lists provided by the best performing teams will be subsequently experimentally evaluated.

In the literature, epitope prediction has been focused primarily on sequence-dependent methods based on various amino acid properties, such as hydrophilicity, solvent accessibility, secondary structure and others [5]–[16]. Several methods based on machine learning approaches have been applied, too [17]. They comprise hidden Markov models (HMM), artificial neural networks (ANN) and support vector machines (SVM) [18]–[22]. Machine learning methods have been frequently coupled with the so-called scale-based approach; this approach exploits one or more scales of amino acid properties to weight each residues of the sequence of interest. In particular it has been shown that the combination of different scales with several machine learning algorithms have better performances than single scale methods [23].

We coped with the DREAM challenge by resorting to a classical supervised machine learning strategy with knowledge-based feature construction. After the definition of the problem features, we developed a logistic regression classifier that showed a very good performance on the test set.

Moreover, we developed a new method for dealing with the bonus round challenge and we generated a list of de-novo peptides that will be further experimentally assessed.

## Materials and Methods

### Data sets

As mentioned in the introduction, one of the DREAM 5 challenges dealt with the prediction of the reactivity of peptides to bind intravenous immunoglobulin (IVIg) antibodies. The challenge organizers made available a dataset that comprises sequences of peptides, which either bind IVIg antibodies with high affinity/avidity or not.

In particular 75534 peptides were incubated with commercially available human IVIg fractions. A set of 6841 peptides with high affinity was identified (positive set). From the same original set, 20437 peptides were identified showing no antibody binding activity in any of the triplicate assays (negative set). Each of these peptides is unique in terms of its amino acid sequence.

Most of these sequences are 15 amino acids long; however, there are also sequences with different lengths (several of them were 13 amino acids long, while a few were long 9, 16, 18, 20 and 21 amino acids).

A reactivity value was calculated for each peptide. The reactivity values range from 1 to 65536. The reactivity of the positive peptides ranges between 10000 and 65536, while this value ranges from 1 to 1000 in the negative peptides case. The training and test datasets were assembled from these two peptide sets.

#### Training set

The training set contained 13638 peptides and was created by selecting 3420 peptides from the positive set and 10218 peptides from the negative set. Two features of each peptide were provided: the amino acid sequence and a measure of the peptide reactivity to the IVIg antibodies. The predictive model of the peptide reactivity was trained on this dataset.

#### Test set

The test set contained 13640 peptides and was formed by grouping the remaining 3421 positive peptides and the remaining 10219 negative peptides. Only the sequence of these peptides was provided for the initial phase of the challenge, while their class (positive or negative) was made available to us only when the results of the challenge had been published.

### Main challenge

The main challenge consists of determining whether peptide reactivity with antibodies is strong or weak, i.e., whether a peptide of the test set belongs to the positive or negative set. The goal is therefore to exploit the training set to develop a predictive model, taking into account the available information (e.g., the information on amino acids and protein-protein interactions available in biological databases). Participants are required to submit a ranked list of the peptides in the test set, ordered according to the predicted probability that the peptide belongs to the positive set (predicted reactivity).

We have dealt with this challenge by applying a proper supervised learning pipeline. The approach consisted in feature selection, classification and cross-validation on the training set and finally evaluation of the model on the test set. These steps followed a crucial phase of knowledge-based construction of the initial set of features.

In the following sub-sections, we will describe, step-by-step, the procedure applied to develop and test the proposed predictive model.

#### Feature construction

The construction of a proper set of features is the most important step of the development of a successful predictive model.

In particular, we considered two sets of features for every peptide: the first set is computed from the peptide sequence, while the second set is generated taking into account the entire training set.

The values of all the features have been normalized between 0 and 1.

In order to generate the first set of features, we exploited information about the peptides and the epitopes reactivity.

In more detail, we used the following peptide attributes:

1. The sequence length, i.e. the number of residues of the peptide.
2. The isoelectric point, computed by using the iterative method described by Tiengo et al. [24].
3. The amino acid frequencies (24 features), calculated as the occurrence of each amino acid along the peptide; the four ambiguous amino acid B (asparagine or aspartic acid), X (unspecified or unknown amino acid), Z (glutamine or glutamic acid) and J (leucine or isoleucine) have also been considered.

As mentioned in the introduction, several approaches have been used for epitope prediction; the so-called scale-based approach exploited one or more scales of amino acid properties to weight each residues of the sequence of interest [2], [18], [25]–[28]. The use of multiple scales was essential to predict epitope location reliably, as reported by Blythe et al. [29]. Therefore, we considered some of the most promising amino acid properties reported in these studies, by resorting to a set of widely used scales (i.e. the five scales reported in Table 1) [9]–[13]:

1. The antigenicity was calculated as proposed by Kolaskar et al. [9]. The frequency of the residue in antigenic determinants (experimentally identified) was exploited to calculate the antigenic propensity of each amino acid.
2. The accessibility was calculated on the basis of the scale proposed by Janin et al. [10]. The importance of the accessibility information is widely reported in the literature; the hypothesis is that an accessible site is likely to be recognized by the antibodies [25], [30]–[32].
3. The hydrophilicity was computed following the scale proposed by Parker et al. [11]. This scale was recently found to have slightly better results than the other ones [2], [33]. The hypothesis for hydrophilicity is that the antigenic sites are on the surface, so they are probably hydrophilic [5], [11].
4. The flexibility was calculated with the scale proposed by Bhaskaran et al. [12]. A high flexibility of the structure is hypothesized to favor the propensity of a peptide to bind the antibodies [34]–[35].
5. The beta-turn prediction was calculated by exploiting an amino acid scale of propensities following the Chou-Fasman method [2], [8], [13].

**Table 1:** Five amino acid scales used for the features construction.

	Antigenicity	Accessibility	Hydrophilicity	Flexibility	Beta-turn
A	1.064	6.6	2.10	0.36	0.66
C	1.412	0.9	1.40	0.35	1.19
D	0.866	7.7	10.00	0.51	1.46
E	0.851	5.7	7.80	0.50	0.74
F	1.091	2.4	−9.20	0.31	0.60
G	0.874	6.7	5.70	0.54	1.56
H	1.105	2.5	2.10	0.32	0.95
I	1.152	2.8	−8.00	0.46	0.47
K	0.930	10.3	5.70	0.47	1.01
L	1.250	4.8	−9.20	0.37	0.59
M	0.826	1.0	−4.20	0.30	0.60
N	0.776	6.7	7.00	0.46	1.56
P	1.064	4.8	2.10	0.51	1.52
Q	1.015	5.2	6.00	0.49	0.98
R	0.873	4.5	4.20	0.53	0.95
S	1.012	9.4	6.50	0.51	1.43
T	0.909	7.0	5.20	0.44	0.96
V	1.383	4.5	−3.70	0.39	0.50
W	0.893	1.4	−10.00	0.31	0.96
Y	1.161	5.1	−1.90	0.42	1.14

Columns 2–6 report five of the most promising amino acid properties for predicting the peptide reactivity: antigenicity [9], accessibility [10], hydrophilicity [11], flexibility [12] and predicted beta-turn propensity [13].

The five attributes described above were computed on the basis of the correspondent amino acid scale, computing the maximum value within a sliding window of 9 residues. The size of the sliding window was chosen because it is known that the binding site covered by an antibody typically includes a stretch of 8 to 10 amino acids [36]–[37].

The second set of features has been generated taking into account the entire training set. To obtain such features, every peptide was aligned with all the others by both the Needleman-Wunsch algorithm (global alignment) and the Smith-Waterman algorithm (local alignment) [38]–[39]. In this way, a scoring matrix [13638×13638] has been computed. In this way, we have generated a set of additional features, as follows:

1. Global alignment. For every peptide we computed: the maximum score obtained by the global alignment with every negative peptides (MaxScore0_nw); the maximum score obtained by the alignment against the positive set (MaxScore1_nw); the difference between MaxScore1_nw and MaxScore0_nw (DiffMaxScore_nw).
2. Local alignment. For every peptide we considered the maximum score of the local alignment with the elements of the positive set and with the elements of the negative set (MaxScore0_sw, MaxScore1_sw), and the difference between these maximum values, as well (DiffMaxScore_sw).

The rationale for selecting the features mentioned above is related to the so-called classification for homology (sequence similarity), which consists of classifying a sequence (in terms of structure and function) looking at the most similar sequence in a dataset of available sequences [40]–[41]. The principle is that similar sequences have similar structures and, thus, similar functions (in this case similar reactivities to antibodies) [42].

In our case, for example, a peptide has a high value of MaxScore0_nw, if the negative examples contain at least another very similar peptide. Moreover, the MaxScore feature is used to check the importance of the absolute value of a good alignment, while the DiffMaxScore attribute takes into account the difference between class groups.

It is important to notice that the use of the information about the class (i.e. positive or negative example) during the feature generation phase requires to properly designing the cross-validation phase in order to avoid overfitting.

Finally, the two types of alignments have been used to understand whether the reactivity depends on the entire sequence of the peptide (global alignment) or on a small portion (local alignment), as hypothesized.

#### Feature selection

Because the training set was made of 13638 examples and the generated features were 37, a features selection step was not mandatory. However, we decided to filter the features to obtain a more parsimonious model. We resorted to a filtering strategy because the use of wrapper methods would have made the cross-validation approaches (and in particular the leave-one-out strategy) computationally very demanding. We have applied three different procedures for feature selection, thus obtaining three different subsets of features.

- *Subset A.* No feature selection - the 37 features generated so far are used.
- *Subset B.* Feature selection with the M5 method [43]–[44]; before applying this approach, all the collinear attributes have been eliminated.
- *Subset C.* Feature selection with the LASSO method (least absolute shrinkage and selection operator) [45].

#### Cross-validation of the classifiers

As mentioned above, the final aim of this challenge is to discover whether there exist rules that enable to predict that a peptide/epitope sequence is recognized by human antibodies. For this reason, we mainly considered classifiers that provide a predictive model easy to be interpreted.

- *Linear regression.* Even if linear regression is a simplistic model due to its strong assumptions, it gives the possibility to evaluate the contribution of each single variable to classification. The outcome variable we considered is the reactivity value, which ranges from 1 to 65536. The distribution of these values shows that the outcome can be easily binarized: in fact, as previously mentioned, the reactivity of the positive peptides ranges between 10000 and 65536, while this value ranges from 1 to 1000 in the negative peptide case. For this reason, we also tested this classifier by considering the binary classes 0-negative and 1-positive as continuous values.
- *Logistic regression.* Also this approach allows assessing the contribution of each variable to classification: in fact, the estimated regression coefficients provide an easy way to evaluate the reliability of the model. Moreover there are no assumptions about the probability distribution of the attributes. However, in the model that we have exploited we supposed that they were not strongly correlated.
- *Naïve Bayes.* It is a simple probabilistic classifier based on the Bayes' theorem under the attribute independence assumption, given the class [46]. The model allows an easy interpretation of the results, since each variable can be separately considered. The main limits of this approach are the strong assumptions of conditional independence between variables and the need of choosing prior distributions.
- *Decision tree.* This method has the great ability to learn complex and non-linear relationships between variables and outcome. Decision trees, however, require the implementation of careful strategies in order to avoid overfitting. In particular, we used the J48 algorithm, an open source Java implementation of the C4.5 method [47]; the dimension of the tree was limited by fixing the minimum number of instances for each leaf equals to 1% of the training set.
- *Rules learner.* This method permits, like decision trees, to extract complex rules; however the accuracy of the predictions is high only if the rules have a sufficiently large support. Moreover, it can be computationally demanding in case of large datasets. In this work we applied the PART method to generate a decision list. Such method is based on an iterative strategy. In each step, PART builds a partial decision tree and converts the best leaf into a rule [48]. The minimum number of instances for each leaf was fixed at 1% of the examples in order to limit the number of generated rules.

To evaluate the best classifier, the performances have been assessed applying the so-called “leave-one-out” cross-validation approach. This approach is particularly suited in our case, since, together with maximizing the size of the training set, it allows to properly generating the features related to the alignment scores.

#### Choice of final model and its interpretation

The model was assessed not only in terms of its predictive performance but also taking into account its interpretation, i.e. by considering the contribution of the different features included in the prediction.

Together with standard performance measures, such as accuracy, sensitivity and specificity, we also computed the F-measure of the predictive model. The F-measure is the harmonic mean of precision (positive predictive value) and recall/sensitivity. As a matter of fact, in order to develop a model that is useful to generate new reactive peptides, it is important to maximize both precision and sensitivity: it means to have a high probability that the peptide predicted to be positive is really reactive and that the reactive peptides are correctly classified.

As previously mentioned, we decided to select, among the best classifiers, the model with the clearest interpretation. In the case of logistic regression, we evaluated the reliability of the regression coefficients by comparing their values and signs with what was expected in the light of the available knowledge.

#### Evaluation of the model and of the teams in the DREAM 5 challenge

As mentioned in the previous sections, the classifiers have been trained on the entire training set. The selected model was then applied on the test set (3421 positive and 10219 negative peptides).

The predictions of all the participants to this DREAM5 challenge have been evaluated and compared. Teams were ranked according to their performance score based on two metrics: the area under the precision versus recall (PR) curve and the area under the receiver operating characteristic (ROC) curve. P-value was defined as the probability that a given or larger area under the curve value is obtained by a random prediction. The overall final score was defined as minus the logarithm of the geometric mean of the ROC and PR p-values.

### Bonus round

The final aim of this challenge is to discover whether there exist rules able to predict reactivity of peptides with human antibodies. These rules can be used to develop new reactive peptides. The “bonus round” was conceived to test the rules learned during the main challenge: each team was required to submit a list of de-novo peptides generated using their predictive models; the list generated by the teams that achieved the top performance in the main challenge will be experimentally validated by the DREAM5 organizers.

In particular, the bonus round challenge required the provided list to contain peptides with sequence length equal to 15, which must follow these specifications:

- at least 1000 peptides in the list should be predicted to have high reactivity, i.e. they should be as reactive as the peptides in the positive training set (high reactivity - H);
- at least 1000 peptides in the list should be predicted to have low reactivity, as the peptides in the negative training set (low reactivity - L);
- at least 1000 peptides in the list should be predicted to have reactivity values in between those of the positive and negative sets (medium reactivity - M).

Moreover, in order to ensure that the peptides of the generated list are different from the peptides of the training and test sets, the following conditions must hold:

1. All submitted peptide sequences should not have stretches of more than three amino acids in common with any of the amino acid sequences supplied in the training or test set.
2. The overall identity between any peptide sequence of the predicted peptides and the training set should not be higher than 5 within a stretch of 11 amino acid positions.

**Figure 1:**
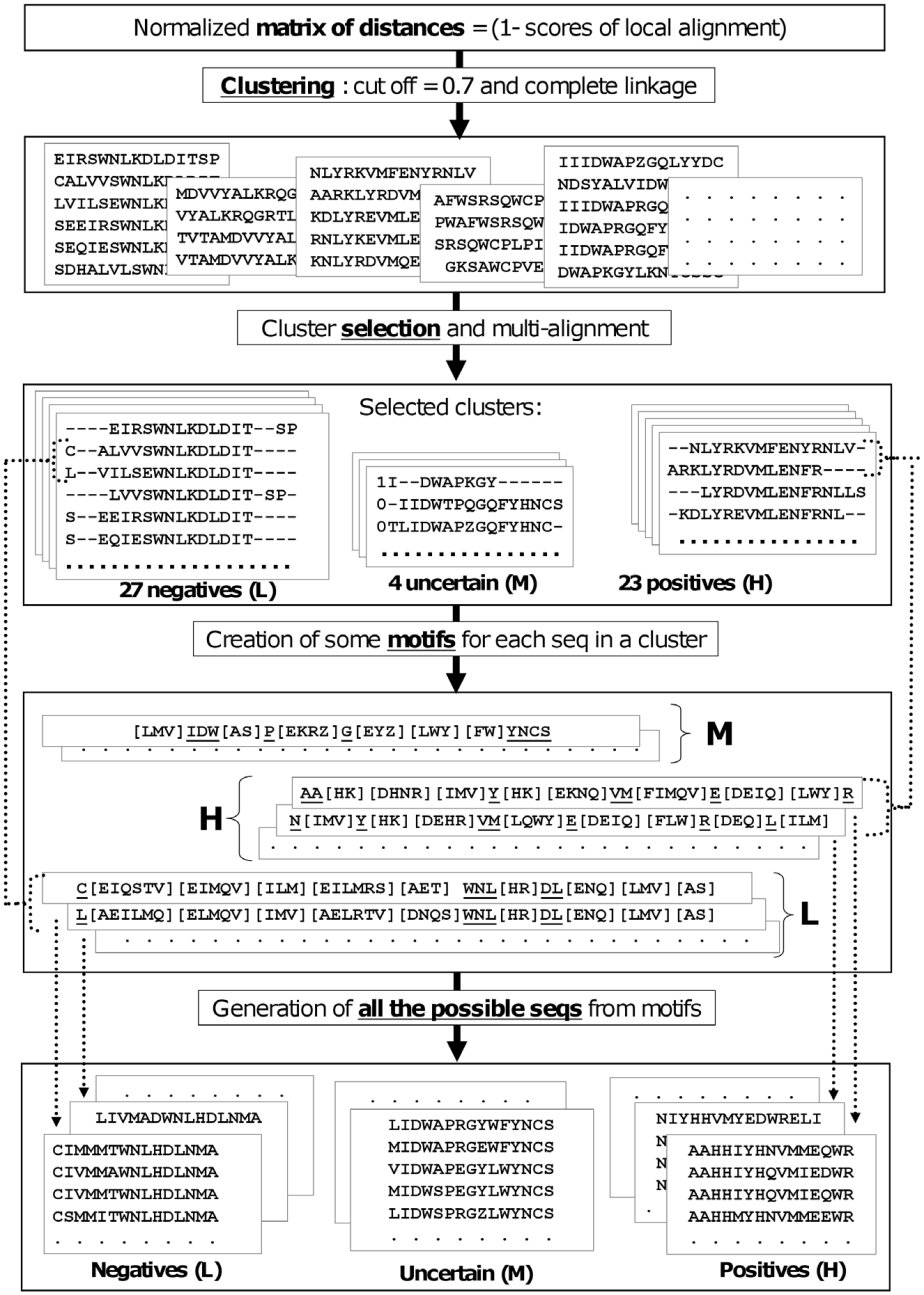
A schematic representation of the procedure for bonus round. The schema shows the principal steps implemented for generating the list of de-novo peptides with low (L), medium (M) and high (H) reactivity: (i) clustering of peptides based on the matrix of distances, (ii) cluster selection and multi-alignment, (iii) creation of some motifs for each sequence in a cluster, (iv) generation of all the possible peptides (followed by the final selection of the peptides based on final model).

In summary, the final output of the bonus round should be a list of 1000 peptides for each of the three classes (i.e. H, L and M). In the next paragraph we describe the procedure we implemented to generate such a list. The main idea is to generate de-novo peptides by extracting from the training set the motifs that characterize the epitope. A schematic representation of the implemented procedure is shown in Figure 1.

#### Clustering

The first step of our strategy is to obtain clusters of similar peptides. In particular we exploited the scoring matrix computed by aligning every sequence with all the others with the Smith-Waterman algorithm (local alignment). We chose local alignment because the results of the main challenge showed that it has higher predictive performance than the global one (see Results). We obtained a distance matrix by subtracting each element of the normalized scoring matrix to one. Then, we applied hierarchical clustering with complete linkage and we used a cut-off value equal to 0.7 to generate the clusters.

#### Cluster selection and multiple-alignment

We selected three types of clusters by exploiting the information about the peptides reactivity.

1. Positive clusters (H) - The clusters with at least five sequences and where all the members are positives.
2. Negative clusters (L) - The clusters with at least eight sequences and where all the members are negative.
3. Uncertain clusters (M) - The clusters with at least five sequences and where the percentage of positive members is similar to the proportion of positive peptides in the training set (3240/13638 = 25%).

A multiple-alignment was then performed on the sequences of each cluster. Thanks to this strategy it was possible to compute the conservation of each amino acid in a specific position.

#### Extraction of the motifs in a cluster

**Figure 2:**
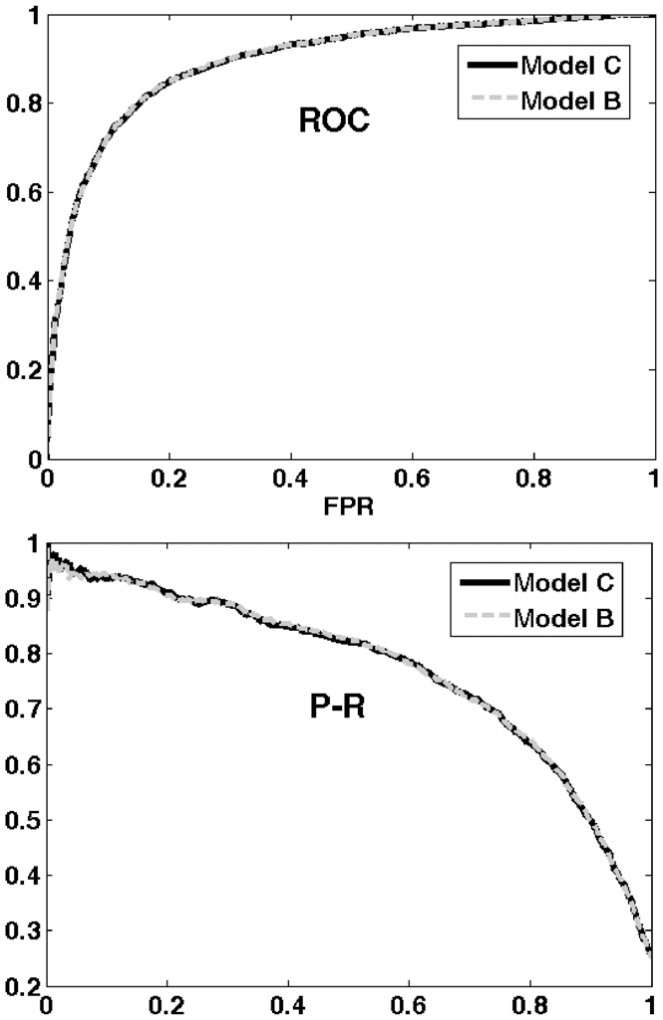
Two examples of peptide clusters. The figure shows two examples of a positive cluster (top) and a negative cluster (bottom). Each cluster of peptide is described by its multiple alignments (on the right top of each sub-figure) and by its representation through sequence logo [49]. This graphical representation displays the conservation of the amino acids in each position of the multi-alignment by their one-letter code. Different residues at the same position are scaled according to their frequency. In particular the height of the entire stack of residues is the information measured in bits (y-axis).

We generated a motif for every sequence 15 amino acids long and belonging to each cluster/multiple-alignment. In detail, we considered all the amino acids composing each of these sequences ordered by the conservation in the corresponding multiple-alignment (computed in terms of information as shown in Figure 2). A residue was kept as constant in the motif if it satisfied the first constraint of the bonus round (no more than three consecutive amino acids already present in the training set). The remaining amino acids are less conserved and do not satisfy the constraint of the bonus round; so these residues were allowed to vary within their amino acid group or following the variation patterns in a specific position reported in the multiple-alignment results. The amino acids groups were obtained by clustering amino acids on the basis of the BLOSUM50 matrix. A motif was thus generated for every sequence in the clusters.

#### Generation of all the possible peptides and selection based on final model

All the possible sequences have been generated starting from the motifs extracted with the method described in the previous paragraph. Such new sequences were then filtered in accordance with the second constraint of the bonus round (identity with the other sequences not higher than 5 amino acids in a window of 11).

The predictive model used in the main challenge (model B) was exploited to predict the reactivities of the remaining new peptides. This prediction has been used to rank the new peptides in terms of predicted reactivity.

We selected the 1100 peptides with the highest predicted reactivity generated from the positive clusters and the 1100 with lowest predicted reactivity obtained from the negative clusters. Finally, we randomly selected 1100 elements from the uncertain clusters.

## Results

### Main challenge

#### Feature selection

As described in the previous section, we generated 37 features to predict peptide reactivity to human antibodies. We applied three different procedures for feature selection: no selection (subset A), selection based on collinear attribute elimination and on the M5 method and (subset B) and selection based on the LASSO method (subset C). The subset B and C contain 28 and 27 remaining attributes, respectively. The subsets B and C are partially different (see Table 2).

**Table 2:** The impact on the predictive performance of the features and the coefficients of the two best models.

	SRCs	F-measure	Log.Reg. B	Log.Reg. C
*LengthSeq*	0.51	41.0%	4.75	−6.97
*Isoel. point*	0.24	33.2%	0.78	0.13
*A*	−0.44	37.1%	7.62	0.00
*C*	0.37	40.1%	6.05	0.77
*D*	−0.25	36.2%	5.79	2.33
*E*	−0.98	41.1%	7.40	0.28
*F*	0.91	45.7%	9.43	−1.91
*G*	0.00	30.3%	8.24	4.35
*H*	0.52	35.2%	8.74	1.49
*I*	−0.04	33.5%	3.48	0.00
*K*	−0.17	34.3%	6.52	2.07
*L*	0.13	31.6%	9.17	0.22
*M*	0.35	35.2%	5.74	2.09
*N*	−0.19	36.9%	3.11	2.08
*P*	−0.26	34.3%	7.01	−0.46
*Q*	−0.43	38.9%	3.17	0.11
*R*	0.45	40.5%	7.78	−1.56
*S*	−0.51	33.1%	8.00	2.59
*T*	−0.20	34.3%	4.72	−0.25
*V*	−0.11	33.9%	3.77	−0.18
*W*	0.94	40.1%	8.78	−0.43
*Y*	1.44	51.0%	11.51	4.49
*B*	0.00	-	0.00	0.00
*X*	0.75	40.1%	0.00	0.00
*J*	0.00	-	0.00	0.00
*Z*	−0.66	40.9%	−4.55	6.50
*Antigenicity*	0.49	31.6%	1.62	−1.13
*Accessibility*	−0.85	40.8%	0.00	0.00
*Hydrophilicity*	−0.71	42.6%	0.00	−0.35
*Flexibility*	−0.78	38.9%	1.26	0.00
*Beta-turn*	−0.26	27.0%	−0.92	1.66
*MaxScore0_nw*	−0.54	38.8%	0.00	0.00
*MaxScore1_nw*	1.36	45.7%	0.00	−6.66
*MaxScore0_sw*	−0.53	37.9%	−3.96	0.00
*MaxScore1_sw*	1.17	52.5%	4.32	0.81
*DiffMaxScore_nw*	−1.35	53.1%	0.00	0.00
*DiffMaxScore_sw*	−1.28	58.9%	0.00	−0.61
Intercept			−15.76	4.73

The second column shows the correlation coefficients of each feature with the class calculated on the training set (single regression coefficients - SRCs). The third column reports the F-measure values of the single attributes. Columns 4 and 5 display the estimated coefficients of the two best models: logistic regression on subset B and logistic regression on subset C; zero values correspond to removed features.

#### Cross-validation of the classifiers

As explained in the Methods section, we learned five different classifiers on the three features subsets. Cross-validation was performed with a leave-one-out approach. The models obtained by applying decision tree and rules learner are reported in the supplementary material (see Text S1 and Text S2).

Table 3 shows the results obtained in terms of mean accuracy, sensitivity, specificity, precision and F-measure:

- The results of the classifiers are in general quite good. This shows that the generated features contain useful information to predict the peptide reactivity.
- The imbalance between the number of positive and negative examples in the training set (3420 positives and 10218 negatives) partially influences the results: the sensitivity is always lower than the specificity.
- Linear regression and logistic regression show the highest performance, even if the difference between the results of the different classifiers is not statistically significant.
- We tested linear regression by both considering discrete or continuous outcomes. This first alternative always gave the best results.
- The best results are obtained after feature selection (subset B and C). This shows that some redundant information is present in the original set of features.
- In terms of F-measure, the logistic regression had a performance clearly higher than all the others (71.15% and 71.17% on subset B and C, respectively). Moreover, considering the quality of the learned models in terms of their Brier Score, the following results were achieved: Lin.Reg. (reactivity) 0.1412; Lin.Reg. (binary) 0.14249; Log. Reg. 0.10344; Naive Bayes 0.13309; Decision tree 0.13717; Rules learner 0.14258. The model based on logistic regression reached the best results, giving a further demonstration of its robustness.

**Table 3:** Results of the classifiers evaluated by leave-one-out cross-validation.

Features	Classifiers	Acc	Sens	Spec	Prec	F-measure
*Subset A*	*Lin.Reg. reactivity*	85.01%	70.12%	90.00%	70.12%	70.12%
	*Lin.Reg.*	85.21%	70.64%	90.09%	70.46%	70.55%
	*Log. Reg.*	85.15%	70.41%	90.09%	70.39%	70.40%
	*Naive Bayes*	83.84%	67.81%	89.21%	67.77%	67.79%
	*Decision tree (J48)*	81.32%	64.56%	86.94%	62.32%	63.42%
	*Rules learner (PART)*	80.75%	63.22%	86.61%	61.25%	62.22%
*Subset B*	*Lin.Reg. reactivity*	84.92%	69.94%	89.94%	69.94%	69.94%
	*Lin.Reg.*	85.17%	70.47%	90.10%	70.43%	70.45%
	***Log. Reg.***	**85.51%**	**71.23%**	**90.29%**	**71.06%**	**71.15%**
	*Naive Bayes*	83.47%	67.08%	88.96%	67.04%	67.06%
	*Decision tree (J48)*	81.40%	63.71%	87.32%	62.71%	63.21%
	*Rules learner (PART)*	77.64%	57.43%	84.40%	55.20%	56.29%
*Subset C*	*Lin.Reg. reactivity*	84.95%	70.00%	89.96%	70.00%	70.00%
	*Lin.Reg.*	85.19%	70.59%	90.08%	70.42%	70.50%
	***Log. Reg.***	**85.54%**	**71.17%**	**90.35%**	**71.17%**	**71.17%**
	*Naive Bayes*	83.02%	66.14%	88.67%	66.14%	66.14%
	*Decision tree (J48)*	81.42%	65.50%	86.75%	62.33%	63.87%
	*Rules learner (PART)*	81.47%	63.28%	87.56%	63.00%	63.14%

The table shows the results of the six classifiers evaluated by leave-one-out cross-validation on three different subsets of features (A, B and C): (i) linear regression considering the reactivity values; (ii) linear regression considering the binary classes 0-negative and 1-positive as continuous values; (iii) logistic regression; (iv) Naïve Bayes; (v) decision tree; (vi) rules learner. The second column displays the name of the tested classifiers. Columns 3–7 report the results reached by each classifier in terms of mean accuracy (Acc), sensitivity (Sens), specificity (Spec), precision (Prec) and F-measure ((2*Sens*Prec)/(Sens+Prec)). The two models with highest F-measure are highlighted in bold.

#### Choice of the final model

The logistic regression models obtained by considering feature subsets B and C have been evaluated in terms of their explanation capabilities.

First of all, we analyzed the two subsets of features by giving some explanations about the removed attributes (zeros are assigned to removed attributes in Table 2, columns 4 and 5).

- By calculating the correlation among the features along the examples and also among the amino-scales used, we found that accessibility, flexibility and hydrophilicity were quite correlated. This is probably the reason why only flexibility was selected in subset B while only hydrophilicity was kept in subset C.
- Concerning the features derived from the alignments, DiffMaxScore attributes were based on MaxScore features, so they are functionally related. The DiffMaxScore feature was removed in subset B because it did not bring any additional information.
- Moreover in subset B all the features derived from global alignment have been removed, suggesting, as expected, that the reactivity depends on a small portion of the peptide, which probably corresponds to the binding site (information retrieved by local alignment). Also in the case of subset C, two out of the three remaining alignment-based features have been derived from local alignment.
- As the amino acid frequencies are concerned, the attributes associated to the presence of X, B and J are present in both subsets B and C, since few sequences contain such residues. The features related to Alanine and Isoleucine have been removed only from subset C.

We then analyzed the estimated coefficients of the logistic regressions in order to further investigate which was the most reliable between the two models. In particular, the estimated coefficients of both models are reported in Table 2, columns 4 and 5. These coefficients have been evaluated on the basis of the available knowledge but also on the basis of the correlation of each feature with the class, as computed in the training set (Single Regression Coefficients - SRCs). The second column of Table 2 reports the regression coefficient computed for each attribute, while column 3 reports its F-measure.

- Every SRC corresponding to an alignment-based feature has the expected sign, given its definition: SRCs are negative for MaxScore_0 and DiffMaxScore features, while they are positive for MaxScore_1. This is confirmed by the corresponding values of the regression coefficients for model B (see Table 2 at column 4). However, model C has an unexpected negative value related to MaxScore_1_nw (see Table 2 at column 5)., Both models confirm that local alignment is more useful for classification than global alignment.
- Concerning the scale-based features, only antigenicity has a positive SRC, as expected. All the other scale-based features have negative SRCs; as a matter of fact, it is known that accessibility, hydrophilicity and flexibility have negative correlations with the reactivity, while it is more difficult to interpret the negative correlation of beta-turn propensity. Both model B and C include only three scale-based features. Given the SRCs and their plausible explanations, both models have only an unexpected regression coefficient, in correspondence of flexibility (model B) and antigenicity (model C).
- No well-defined knowledge is available about the influence of the amino acid frequencies on the peptide reactivity. So, we assessed the correlation of the corresponding SRCs with the different amino acid scales (antigenicity: 0.29; accessibility: −0.66; hydrophilicity: −0.69; flexibility: −0.52; beta-turn propensity: −0.19). The sign of the correlations (i.e. Spearman correlation) follows the same pattern of the scale-based features: all the features are negatively correlated with peptide reactivity except for antigenicity.Finally, we evaluated the reliability of the regression coefficients of the multivariate model in terms of their correlation with the SRCs. Model B showed a good positive correlation between the regression coefficients and the correspondent SRCs (i.e. Spearman correlation = 0.496 and p-value = 0.028), while in model C no correlation was found (i.e. Spearman correlation = −0.075 and p-value = 0.753).

Based on all these considerations, we selected model B as the best final model, even if model C had a higher F-measure.

#### Evaluation of the model

The selected model was used to generate predictions on the test set data (3421 positive and 10219 negative peptides). The predictions of our model, as well as of the other participants to DREAM5 challenge 1, were evaluated in terms of a score based on the area under the precision versus recall (PR) curve and the area under the receiver operating characteristic (ROC) curve.

**Figure 3:**
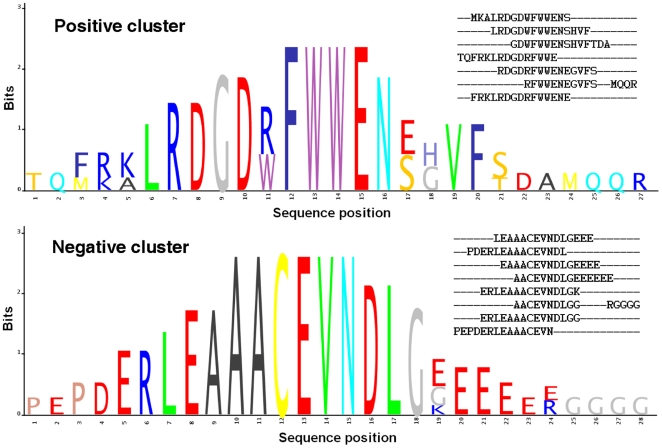
ROC curve and Precision-Recall curve of the two best final models. The figure displays the ROC curve (top) and the Precision-Recall curve (P-R) (below) calculated for the two best final models: logistic regressions fitted on the features contained in the subset B (model B) and in the subset C (model C).

The results obtained by Model B and Model C are reported in Table 4. Both models had a very high performance in terms of PR and ROC, as shows in Figure 3. It is important to note that Model B achieved the highest score.

**Table 4:** Scores of the participants to DREAM5 challenge 1.

Team	AUPR	AUROC	Pval AUPR	Pval AUROC	Score
*Team 725*	0.772	0.893	2.05E-23	4.75E-19	20.51
***Log. Reg. B***	**0.768**	**0.895**	**3.20E-22**	**1.00E-19**	**20.25**
***Log. Reg. C***	**0.767**	**0.894**	**4.11E-22**	**1.48E-19**	**20.11**
*Team 161*	0.691	0.864	4.08E-06	1.26E-08	6.64
*Team 763*	0.689	0.855	6.56E-06	4.02E-06	5.29
*Team 795*	0.678	0.850	1.76E-04	4.91E-05	4.03
*Team 852*	0.663	0.849	5.92E-03	9.20E-05	3.13
*Team 730*	0.662	0.846	6.59E-03	3.86E-04	2.80
*Team 809*	0.636	0.846	2.79E-01	3.46E-04	2.01
*Team 834*	0.597	0.835	9.89E-01	1.85E-02	0.87
*Team 433*	0.627	0.803	5.71E-01	9.85E-01	0.13
*Team 703*	0.596	0.813	9.91E-01	8.19E-01	0.05
*Team 811*	0.604	0.748	9.70E-01	1.00E+00	0.01
*Team 528*	0.565	0.790	1.00E+00	1.00E+00	0.00
*Team 550*	0.355	0.612	1.00E+00	1.00E+00	0.00
*Team 737*	0.582	0.793	9.99E-01	9.99E-01	0.00

The table shows the performance of all the participants to DREAM5 challenge 1. Columns 2–5 displays the Area under the Precision-Recall curve (AUPR), the Area under the ROC curve (AUROC), the p-value of AUPR (Pval AUPR) and p-value of (Pval AUROC), respectively. All the participants are evaluated in terms of the final overall score (reported in column 6); it was defined as minus the logarithm of the geometric mean of Pval AUROC and Pval AUPR. The results of our two best models are highlighted in bold.

By analyzing the scores of all the participants, reported in Table 4, it can be noted that two teams (our team and team 725) clearly over-performed all the others.

### Bonus round

As explained in the previous section, the final output of the bonus round is a list of 1000 new peptide sequences for each of the three classes: high reactivity (H), low reactivity (L) and medium reactivity (M).

The procedure for the generation of these peptides follows the steps described in the Methods section and schematically reported in Figure 1.

In the first phase we used the scores of local alignment to cluster the available sequences. As result of this first phase, about 7000 clusters with different size have been created.

Then we exploited the class information to select three types of clusters. By applying the rules described in the Methods section, we selected 23 positive clusters, 27 negative clusters and 4 uncertain clusters. An example of positive cluster and an example of negative one are shown in Figure 2: the figure depicts the multiple alignments of the two clusters and their representation through sequence logos [49].

The motif generation phase resulted in a few thousands sequences for each class group (i.e. H, L and M).

**Figure 4:**
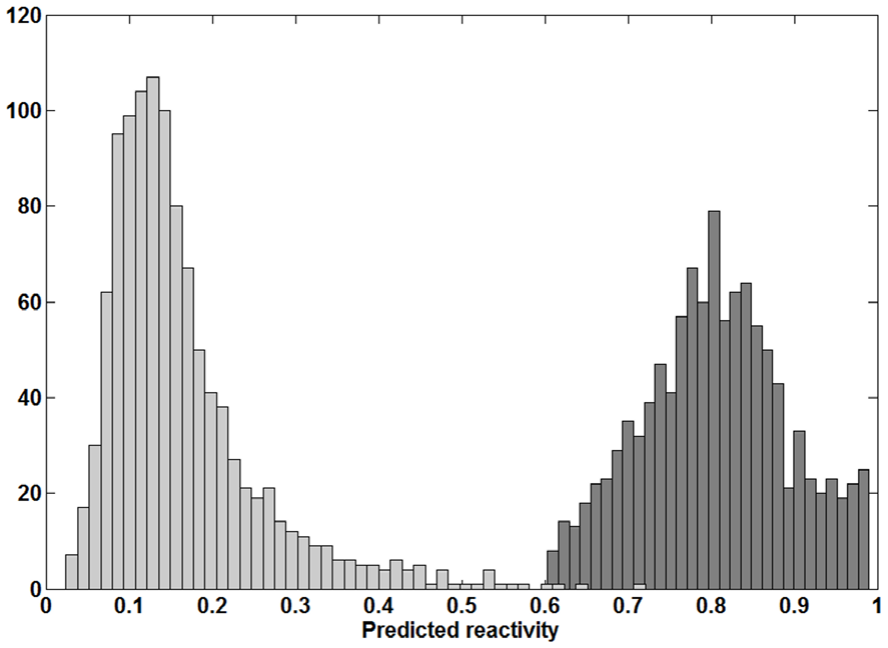
The distributions of the predicted reactivities of bonus round peptides. We selected the same number of peptides (i.e. 1100) both deriving from negative and from positive clusters. The reactivity of the peptides of the two groups is predicted through the final best model proposed for the main challenge. This figure shows the histogram of such predicted reactivities. In particular we considered the binary classes 0-negative (reactivity between 1 and 1000) and 1-positive (reactivity between 10000 and 65536); the predicted reactivity of a peptide in the range [0∶1] is given by the probability to belong to the positive class.

Finally, we computed the predicted reactivity for all the sequences generated from the positive and negative clusters. The final list was formed by: i) the 1100 peptides with the highest predicted reactivity generated from the positive clusters, ii) the 1100 with lowest predicted reactivity from the negative clusters and iii) 1100 peptides randomly selected from the uncertain clusters. As shown in Figure 4, the distributions of the predicted reactivity clearly separate the peptides coming from the positive clusters and the negative ones. This demonstrates the validity of the strategy adopted to generate new peptides.

The experimental test of the real reactivity of these peptide sequences is still ongoing.

## Discussion

In the present work we described the procedure implemented to cope with the Epitope-Antibody Recognition (EAR) Specificity Prediction Challenge of the DREAM5 competition. The aim of the EAR challenge was to extract rules able to predict the binding of a peptide/epitope to a human antibody. A training set of peptides with experimentally identified high and low reactivity to human antibodies was provided. The challenge consists therefore in determining whether the peptides of an independent test set belong to the positive or negative set.

As mentioned in the previous section, we have exploited a machine learning approach to analyze the data, after a knowledge-based feature generation phase. In particular we extracted two types of features for every peptide: (i) sequence-dependent features, which are based on both general information about peptides and knowledge about the propensity of a peptide to interact (amino acid frequencies, antigenicity, accessibility, etc.); (ii) dataset-dependent features, which are generated by exploiting the scores obtained by aligning every peptide of the training set with all the others with both global and local alignment. A total of 37 features have been finally generated.

We considered three different subsets of such attributes, based on different feature selection strategies. As a last step, we learned some simple classifiers, which have been evaluated with a leave-one-out cross-validation approach. Since the final aim of the EAR challenge is to extract rules able to explain the propensity of peptide to react, we selected classifiers able to provide a model easy to be interpreted (e.g. logistic regression, rule learners, decision trees, etc.).

The classifier finally selected was built with the logistic regression, one of the most widely used classifiers, able to predict the probability of the class on the basis of both continuous and discrete features. The best results were achieved by using a reduced subset of features; in particular, taking into account the model interpretation needs, we selected the logistic regression fitted with the features obtained by M5 method.

The evaluation of the prediction of the model on the test set showed the validity of the approach: the model had one of the best performances of the challenge. As a note, the performances of the model on the test set are higher than the one obtained with cross-validation on the training set (e.g. F-measure metrics are 71.26% and 71.15%, respectively).

In general, this good performance has demonstrated that, even if the prediction of epitope reactivity is a difficult problem, there are ways to obtain promising predictive models based on the combination of prior knowledge and data analysis [50].

Together with the high performance of the proposed reactivity prediction model, the present work highlights some open issues concerning the propensity of a peptide to react with human antibodies.

- The features based on local alignment are more predictive than the ones based on global alignment. This shows that, as expected, the reactivity depends on a small portion of the peptide, which probably corresponds to the binding site.
- In contrast to some hypotheses previously formulated, the features related to accessibility, flexibility and hydrophilicity are negatively correlated with the reactivity values of the dataset.Concerning accessibility and hydrophilicity, the hypothesis that the antigenic sites are on the surface, and thus probably hydrophilic, was recently confuted [9]. As a matter of fact, the analysis of the experimentally determined antigenic sites has revealed that the hydrophobic residues are more likely to be a part of antigenic sites if they occur on the surface of a protein.Moreover, some studies hypothesized that the flexibility is inversely proportional to antigenic propensity [9], [51]: a relatively high positive correlation (i.e. Spearman correlation = 0.61 and p-value = 0.018) was found between the flexibility and the minimum concentration needed to inhibit the E.Coli growth with antimicrobial peptides. As a matter of fact, a small flexibility may be related to a compact structure, which could favor antigenic propensity.
- Antigenicity is positively correlated with the reactivity value. This result confirms the appropriateness of the scale used for its calculation [9]: this is defined as the ratio between the frequency of the residue in antigenic determinants (experimentally identified) and the frequency of the amino acid on the surface (predicted by using the average of hydrophilicity, accessibility and flexibility values reported by Parker et al. [11]).
- Finally, the high values of both SRCs and regression coefficients, as shown in Table 2 (see columns 2 and 4), demonstrate that some amino acids, like F and Y, favour the peptide reactivity.

The future developments of this work will concern the test of our model on other datasets related to the prediction of epitope reactivity. Preliminary encouraging results have been achieved on some peptides of the IEDB (Immune Epitope DataBase) [52].

The final aim of the challenge was to elucidate the mechanisms of epitope reactivity with human antibodies; for this reason, a “bonus round” was proposed beside this main challenge. To this end, we have developed a method based on a clustering approach. We grouped the peptides of the training set in about 7000 clusters by using as distance the score of the local alignment. Then, we selected 23 positive, 27 negative and only 4 uncertain clusters by a-posteriori taking into account of the class of the peptides. It is worthwhile mentioning that the small number of negative and positive clusters demostrates that there are many rules underlying peptide reactivity. Each rule has thus a small support; this is probably related to the wide variability of the antibodies.

The clusters have been used to extract a set of motifs that were the basis to generate an initial list of potential new peptides. We predicted the reactivity of such new peptides relying on our model: the sequences with highest and lowest predicted reactivity formed the final list of de-novo peptides. The results of the experimental test of the real reactivity of these peptide sequences will be available in the near future.

## Supporting Information

Text S1 **J48 pruned tree learned on the entire training set starting from the features subset B.**
(DOC)

Text S2 **PART decision list learned on the entire training set starting from the 28 features belonging to the subset B.**
(DOC)
