# Correction: Prediction of Peptide Reactivity with Human IVIg through a Knowledge-Based Approach

**DOI:** 10.1371/annotation/57a9c527-109a-435f-a78d-1b7bce0e578b

**Published:** 2011-09-02

**Authors:** Nicola Barbarini, Alessandra Tiengo, Riccardo Bellazzi

Figure 2 and Figure 3 should be swapped. 

