# Supplementary material for: Prediction of Peptide Reactivity with Human IVIg through a Knowledge-Based Approach
Source: PLoS One. 2011 Aug 24;6(8):e23616. doi: 10.1371/journal.pone.0023616 (PMC3160895; doi:10.1371/journal.pone.0023616)
Supplement: Text S1 — J48 pruned tree learned on the entire training set starting from the features subset B. (DOC) [file pone.0023616.s001.doc]

**Text S1. J48 pruned tree learned on the entire training set starting from the features subset B.**

The shown tree (obtained by Weka 3.7) is composed by 14 leaves and its size is 27. The tree highlights some issue discussed in the manuscript on the relationship features-reactivity: first of all both the remaining features related to local alignment are crucial for classification with opposite contributions; then Tyrosine seems to favor the peptide reactivity, while the flexibility seems to be negatively correlated with the outcome.

| MaxScore1_sw <= 0.30097  | Y <= 0 THEN 0 (7984.0/869.0)  | Y > 0  | | MaxScore0_sw <= 0.333333  | | | lengthSeq <= 0 THEN 0 (408.0/73.0)  | | | lengthSeq > 0  | | | | Y <= 0.203426  | | | | | isoel.point <= 0.602444  | | | | | | flexibility.txt <= 0.477528  | | | | | | | W <= 0 THEN 0 (228.0/92.0)  | | | | | | | W > 0 THEN 1 (177.0/66.0)  | | | | | | flexibility.txt > 0.477528 THEN 0 (810.0/197.0)  | | | | | isoel.point > 0.602444 THEN 1 (388.0/155.0)  | | | | Y > 0.203426 THEN 1 (675.0/217.0)  | | MaxScore0_sw > 0.333333 THEN 0 (975.0/102.0)  MaxScore1_sw > 0.30097  | MaxScore0_sw <= 0.344443  | | Y <= 0.203426  | | | flexibility.txt <= 0.567416 THEN 1 (698.0/140.0)  | | | flexibility.txt > 0.567416  | | | | isoel.point <= 0.593045 THEN 0 (149.0/65.0)  | | | | isoel.point > 0.593045 THEN 1 (148.0/37.0)  | | Y > 0.203426 THEN 1 (327.0/26.0)  | MaxScore0_sw > 0.344443  | | MaxScore1_sw <= 0.60194 THEN 0 (495.0/129.0)  | | MaxScore1_sw > 0.60194 THEN 1 (176.0/55.0) |
| --- |
