# Supplementary material for: Prediction of Peptide Reactivity with Human IVIg through a Knowledge-Based Approach
Source: PLoS One. 2011 Aug 24;6(8):e23616. doi: 10.1371/journal.pone.0023616 (PMC3160895; doi:10.1371/journal.pone.0023616)
Supplement: Text S2 — PART decision list learned on the entire training set starting from the 28 features belonging to the subset B. (DOC) [file pone.0023616.s002.doc]

**Text S2. PART decision list learned on the entire training set starting from the 28 features belonging to the subset B.**

The shown list is composed by nine rules and were obtained by Weka 3.7. The learned rules shows some general tendencies discussed in the manuscript about the relationship between the feature and the outcome: the two features related to local alignment are both important for classification with opposite contributions; then some amino acid like Y and F seems to favor the peptide reactivity; at last the antigenicity feature was positively correlated with the reactivity value.

| - MaxScore1_sw <= 0.30097 AND   Y <= 0 THEN 0 (7984.0/869.0)   - MaxScore0_sw > 0.344443 AND   MaxScore1_sw <= 0.582525 THEN 0 (1419.0/217.0)   - lengthSeq > 0 AND   MaxScore1_sw > 0.165047 AND  Y <= 0.164882 AND  E <= 0.101822  THEN 1 (1274.0/363.0)   - Y > 0.164882 AND   MaxScore1_sw > 0.194173 THEN 1 (504.0/64.0)   - Y <= 0.203426 AND   Y <= 0.143469 AND  isoel.point <= 0.602444 AND  F <= 0.164882 THEN 0 (999.0/264.0)   - lengthSeq > 0 AND   W <= 0 AND  antigenicity.txt > 0.400438 THEN 1 (530.0/190.0)   - lengthSeq <= 0 THEN 0 (413.0/76.0) - W > 0 THEN 1 (296.0/79.0) - ELSE 0 (219.0/86.0) |
| --- |
